# Supplementary material for: The Evolutionary Landscape of SARS-CoV-2 Variant B.1.1.519 and Its Clinical Impact in Mexico City
Source: Viruses. 2021 Oct 29;13(11):2182. doi: 10.3390/v13112182 (PMC8617872; doi:10.3390/v13112182)
Supplement: Supplementary file 1 [file viruses-13-02182-s001.zip › viruses-1426612-supplementary.pdf]

The evolutionary landscape of SARS-CoV-2 variant B.1.1.519 and its clinical impact in Mexico City

SUPPLEMENTARY INFORMATION

SUPPLEMENTARY FIGURES

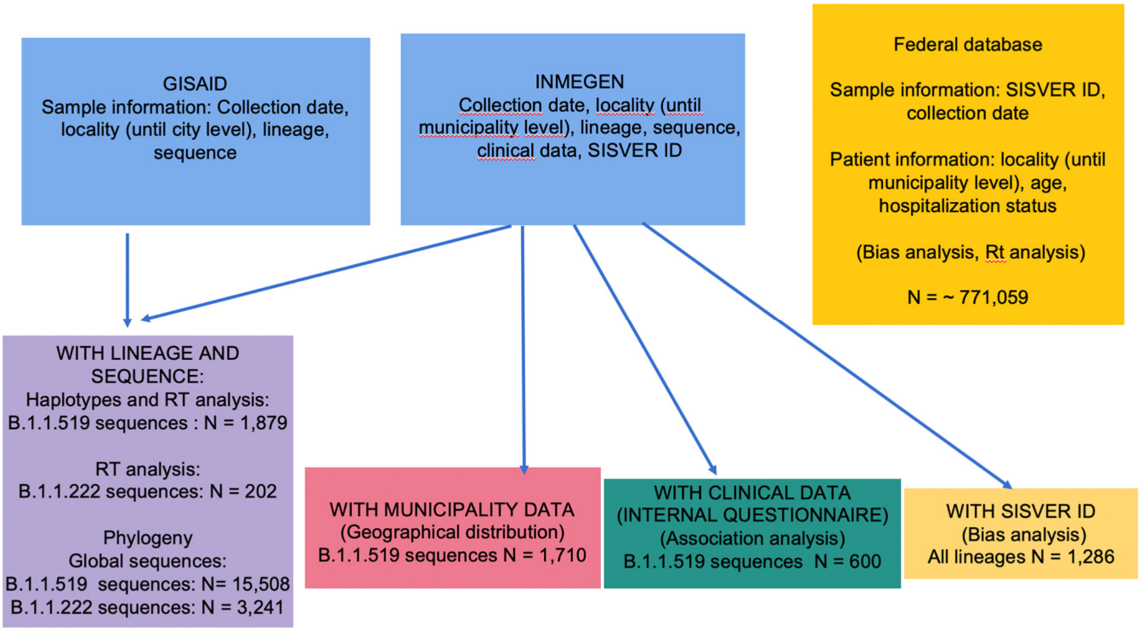

Figure S1. Study design. GISAID and INMEGEN database were the main sources of information. The Rt, haplotype and phylogeny analysis were done using sequences obtained from both sources. Only INMEGEN sequences were used during the geographical distribution, the association and the bias analysis since only INMEGEN samples have municipality level data, comprehensive clinical data and a SISVER ID.

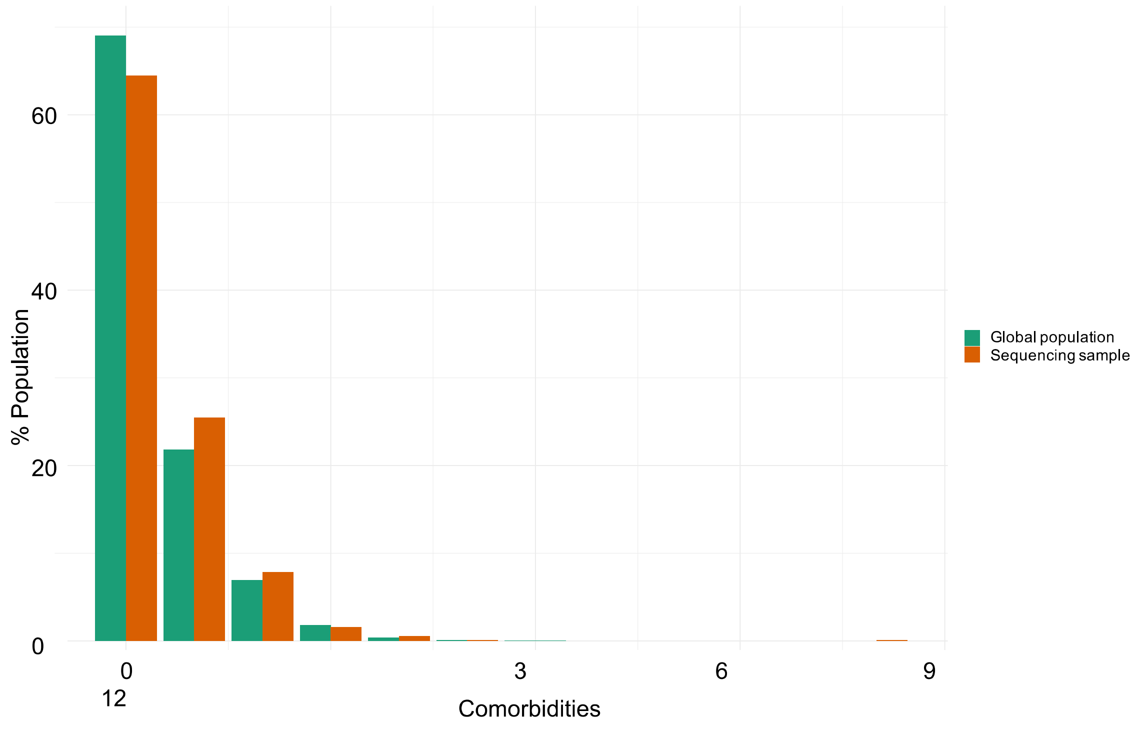

Figure S2. Percentage of population with a given number of comorbidities. The number of comorbidities is represented in the X axis, and the percentage population with that specific number of comorbidities is represented in the Y axis. The color represent sample type: orange corresponds to the samples with sequencing data and a SISVER ID (sequenced at INMEGEN); green corresponds to all other samples in the federal database.

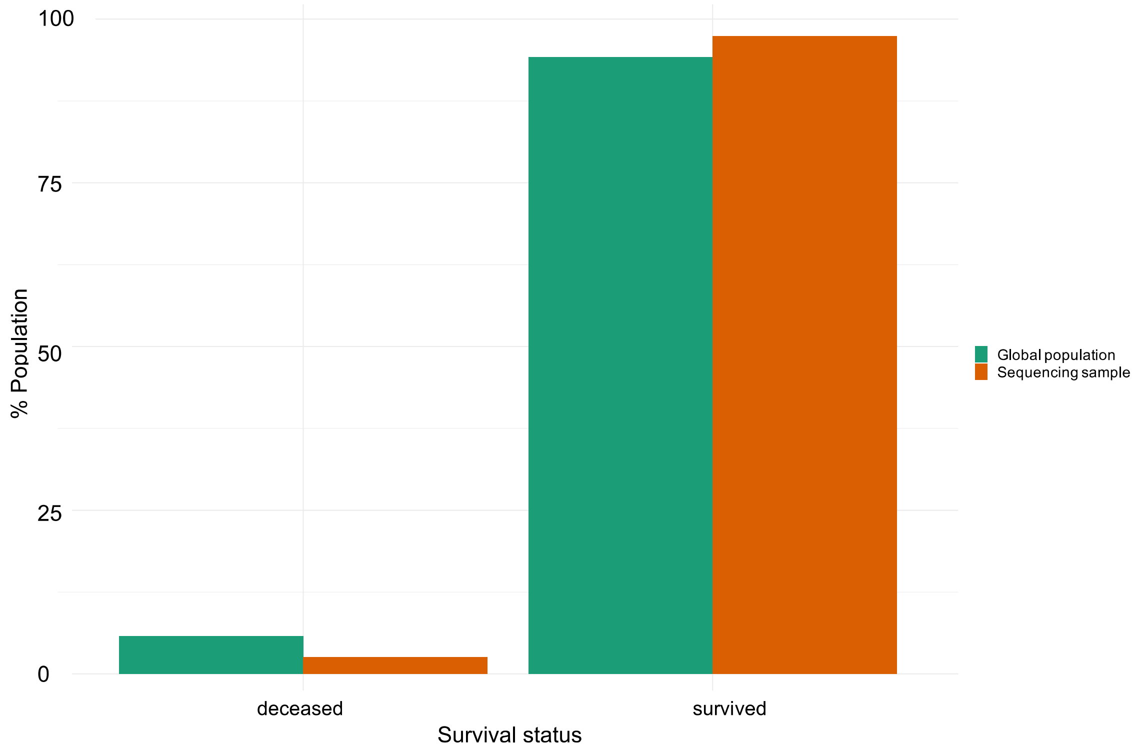

Figure S3. Percentage of population with a given survival status (deceased or survived). The survival status is represented in the X axis, and the percentage population per survival status is represented in the Y axis. The color represent sample type: orange corresponds to the samples with sequencing data and a SISVER ID (sequenced at INMEGEN); green corresponds to all other samples in the federal database.

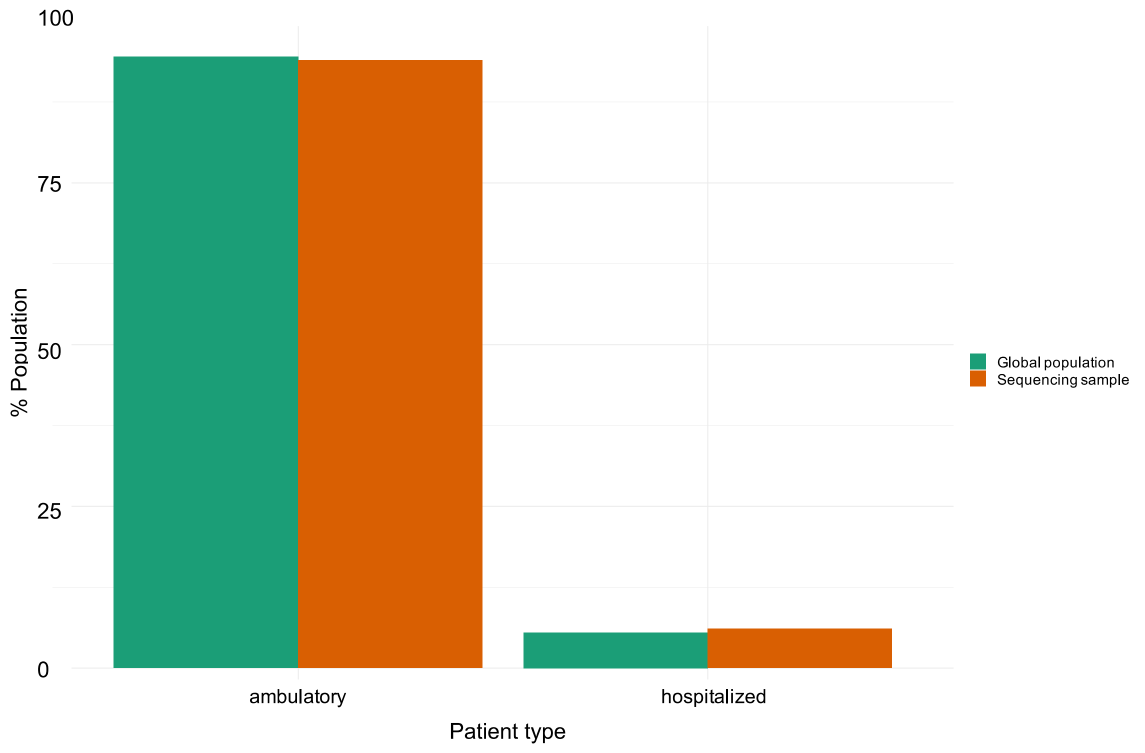

Figure S4. Percentage of population with a given hospitalization status (ambulatory or hospitalized). The hospitalization status is represented in the X axis, and the percentage population per hospitalization status is represented in the Y axis. The color represent sample type: orange corresponds to the samples with sequencing data and a SISVER ID (sequenced at INMEGEN); green corresponds to all other samples in the federal database.

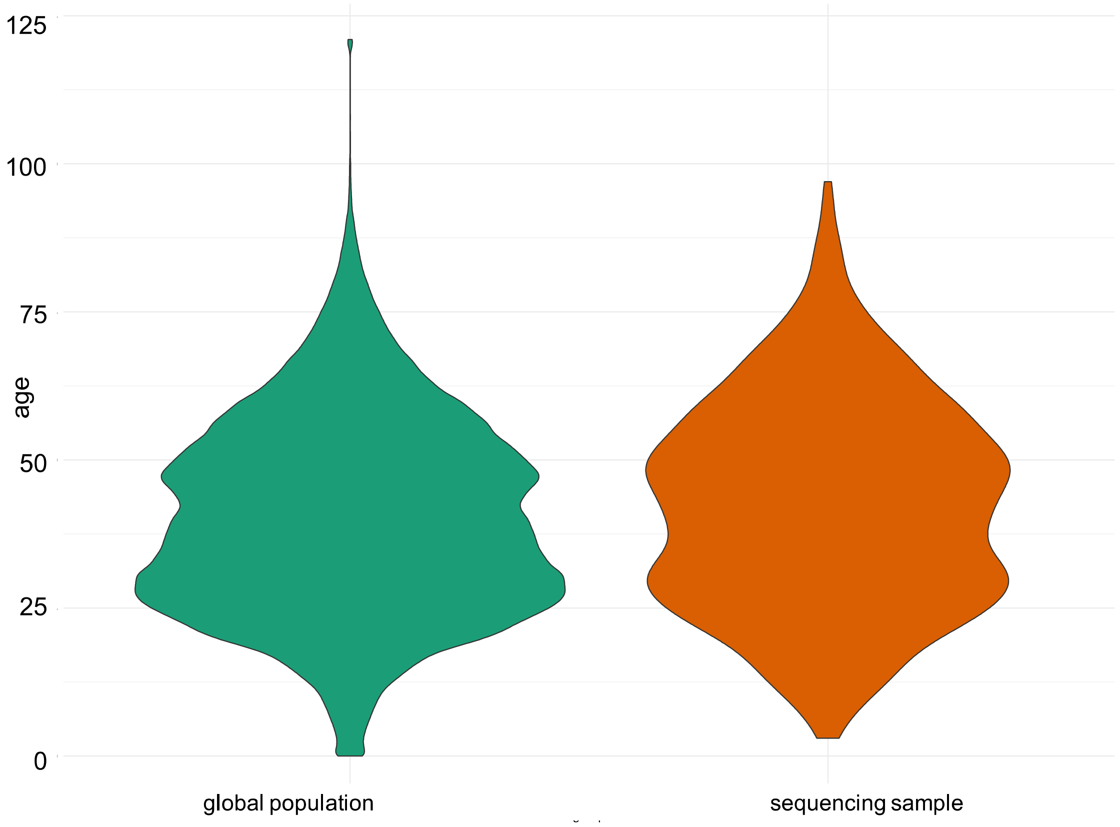

Figure S5. Age distribution per sample type. Orange corresponds to the samples with sequencing data and a SISVER ID (sequenced at INMEGEN); green corresponds to all other samples in the federal database.

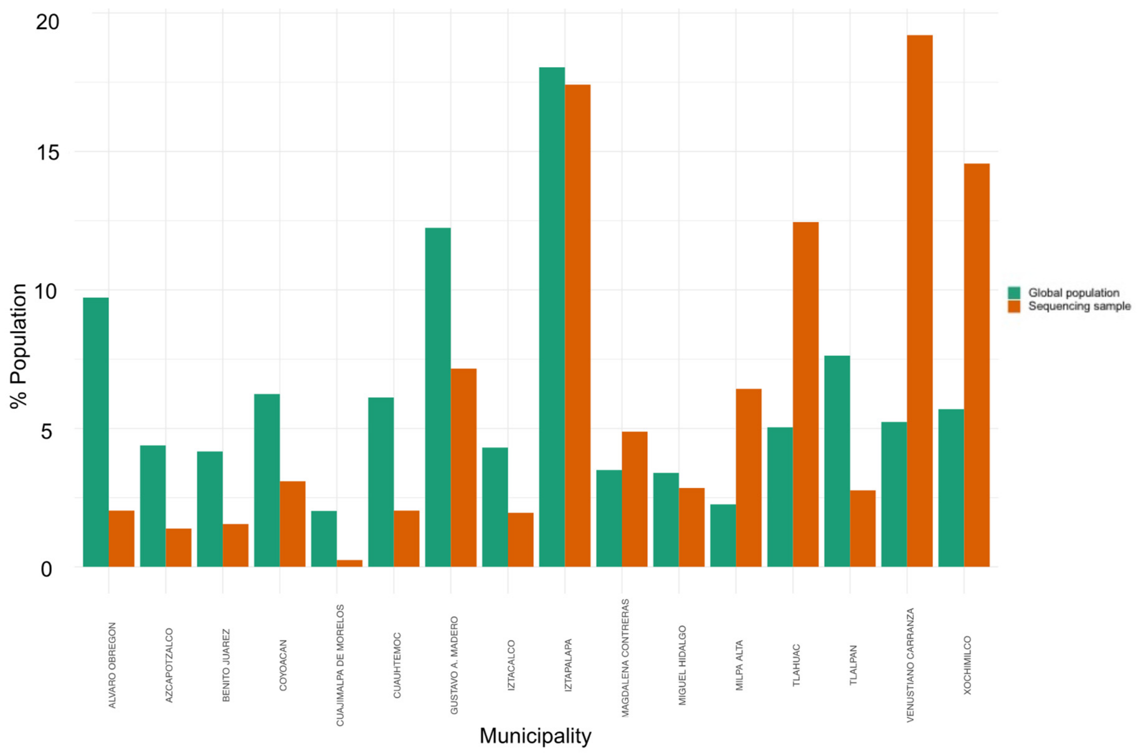

Figure S6. Percentage of population per municipality. The municipality is represented in the X axis, and the percentage population municipality is represented in the Y axis. The color represent sample type: orange corresponds to the samples with sequencing data and a SISVER ID (sequenced at INMEGEN); green corresponds to all other samples in the federal database.

SUPPLEMENTARY TABLES

Symptom	B.1.1.519 n(%)	Other n(%)	aOR	95% CI	p-value
Dyspnea	153 (50.7%)	104 (37.4%)	1.766	(0.187 - 0.956)	0.0037
Cyanosis	20 (6.6%)	9 (3.2%)	3.671	(0.161 - 2.794)	0.0453
Chest.Pain	160 (53.0%)	119 (42.8%)	1.429	(-0.016 - 0.732)	0.0614
Diarrhea	112 (37.1%)	92 (33.1%)	1.451	(-0.019 - 0.770)	0.0640
Polipnea	40 (13.2%)	46 (16.5%)	1.757	(-0.047 - 1.222)	0.0799
Conjuntivitis	68 (22.5%)	90 (32.4%)	0.735	(-0.729 - 0.115)	0.1514
Myalgia	209 (69.2%)	183 (65.8%)	1.303	(-0.124 - 0.651)	0.1807
Odynophalgia	143 (47.4%)	144 (51.8%)	0.793	(-0.606 - 0.139)	0.2205
Rhinorrhea	98 (32.5%)	103 (37.1%)	0.793	(-0.619 - 0.156)	0.2400
Arthralgia	195 (64.6%)	173 (62.2%)	1.250	(-0.156 - 0.602)	0.2480
Anosmia	173 (57.3%)	183 (65.8%)	0.808	(-0.595 - 0.166)	0.2719
Cough	202 (66.9%)	168 (60.4%)	1.203	(-0.202 - 0.569)	0.3475
Persistant.Fever	47 (15.6%)	52 (18.7%)	0.824	(-0.691 - 0.311)	0.4462
Cephalea	211 (69.9%)	203 (73.0%)	0.858	(-0.575 - 0.262)	0.4732
Vomit	30 (9.9%)	27 (9.7%)	1.235	(-0.417 - 0.874)	0.5184
Fever	183 (60.6%)	175 (62.9%)	0.907	(-0.487 - 0.288)	0.6196
Abdominal.Pain	31 (10.3%)	37 (13.3%)	1.062	(-0.546 - 0.688)	0.8487

Table S1. Associations between symptoms and variant B.1.1.519 using multivariate LR adjusted for covariates excluding asymptomatic patients.

	Summary	Ordinal Multivariable LR Model (Severity)			Binary Multivariable LR Model (Hospitalization)		
Characteristic	N = 580[1]	OR[2]	95% CI[2]	p-value	OR[2]	95% CI[2]	p-value
Severity							
Mild	292 (50%)						
Severe	255 (44%)						
Dead	33 (5.7%)						
Hospitalized	69 (12%)						
Age	42 (29, 54)	1.04	1.03, 1.05	**<0.001**	1.06	1.04, 1.09	**<0.001**
Sex							
Female	293 (51%)	—	—		—	—	
Male	287 (49%)	1.21	0.87, 1.70	0.3	1.78	1.00, 3.21	0.053
Ct	19.13 (17.90, 20.40)	0.99	0.93, 1.06	0.8	1.05	0.94, 1.16	0.4
ImmunoSuppressed	18 (3.1%)	2.85	1.12, 7.40	**0.029**	2.41	0.59, 8.15	0.2
HD_Hypertension	103 (18%)	1.18	0.73, 1.90	0.5	1.56	0.82, 2.94	0.2
Diabetes	71 (12%)	0.91	0.53, 1.56	0.7	1.09	0.53, 2.16	0.8
Obesity	229 (39%)	1.41	1.01, 1.98	**0.047**	1.67	0.94, 2.99	0.079
Asthma	20 (3.4%)	1.53	0.62, 3.72	0.4	1.06	0.20, 4.22	>0.9
Smoker	161 (28%)	1.21	0.83, 1.75	0.3	0.85	0.44, 1.60	0.6
Variant							
Other	278 (48%)	—	—		—	—	
B.1.1.519	302 (52%)	1.84	1.32, 2.57	**<0.001**	2.21	1.24, 4.09	**0.0089**

[1]n (%); Median (IQR)

[2]OR = Odds Ratio, CI = Confidence Interval

Table S2. Association of the SARS-CoV-2 B.1.1.519 variant with disease severity and hospitalizations excluding asymptomatic patients. The severity outcomes were coded as 0=Asymptomatic/Mild, 1=Severe, or 2=Dead; an ordinary multivariate LR model was fitted adjusted for covariates. A binary multivariate LR model was fitted for hospitalization.
